# Assessment of quality of essential medicines in public health care facilities of Nepal: Findings of nationwide study

**DOI:** 10.1371/journal.pgph.0001841

**Published:** 2023-05-25

**Authors:** Neelam Dhakal, Pradip Gyanwali, Baburam Humagain, Rajendra BC, Nisha Jha, Phoolgen Sah, Amita Pradhan, Meghnath Dhimal, Anjani Kumar Jha

**Affiliations:** 1 Nepal Health Research Council, Ramshahpath, Kathmandu, Nepal; 2 Department of Pharmacology, Maharajgunj Medical Campus, Kathmandu, Nepal; 3 Management Section for Health (MSH), Kathmandu, Nepal; 4 Department of Clinical Pharmacology and Therapeutics, KIST Medical College and Teaching Hospital, Lalitpur, Nepal; 5 Department of Pharmacy and Pharmacology, Janamaitri Foundation, Institute Of Health Sciences, Kathmandu, Nepal; 6 Department of Community Dentistry, People’s Dental College and Hospital, Kathmandu, Nepal; Jawaharlal Nehru Medical College, INDIA

## Abstract

Essential medicines are those medicines that satisfy the primary health care needs of the citizens. Poor quality of essential medicines can have serious impact on public health. Thus, this study is aimed to assess the quality of essential medicines available in public health care facilities of Nepal. A cross sectional descriptive study was carried out in 62 health facilities across 21 districts, representing all seven provinces of Nepal and selected proportionately from all three ecological regions i.e. Terai, Hill and Mountain using lottery method. Health facilities in selected districts were chosen using random number generator. Face to face interview was taken with health facility in charge using structured questionnaire. All storage conditions information was recorded through observation checklists. Temperature and humidity were measured using a digital instrument. Similarly, 20 different generic medicines were collected for quality testing. The obtained data were entered in Epidata version 3.1, cleaned in Microsoft Excel 2007 and analyzed in SPSS version 16.0. Among 62 health facilities, only 13% of health facilities were found to follow the medicine storage guidelines, with temperature and humidity levels exceeding recommended limits. Out of 244 batches of 20 different generics of essential medicines, 37 batches were found to be substandard. These substandard medicines were- Ciprofloxacin hydrochloride eye/ear drop, Iron supplement tablets, Metformin Hydrochloric tablet, Metronidazole Tablets, Paracetamol Oral suspension, Paracetamol tablet and Povidone Iodine solution. The study recommends the urgent need for the Government of Nepal to prioritize ensuring the quality of essential medicines in the country.

## Introduction

In recent days, there has been a growing concern about the quality of medicines all around the world. Poor quality of medicines which includes substandard, spurious, falsely labeled, falsified and counterfeit medicines (SSFFC) have resulted serious impacts [1] and are a prominent issue in both developed and developing countries [2, 3]. Availability of safe, effective and affordable medicine is one of the indicators of quality of health services [4] and also a major target area of Universal health coverage [5]. However, quality of medicine seems to be neglected in most of the developing countries. There is increased risk for circulation of substandard/counterfeit products worldwide due to globalization; more notably to the developing countries with weak regulatory authorities [6]. Evidences show prevalence of substandard and counterfeit medicines worldwide encompassing drugs of classes: anti-infective, anti-malarial, paracetamol, antibiotics, antihelminthic [2, 7–10].

Nepal is a developing country in South Asia with a population of around 30 million people [11]. The government has established an Essential Medicines Program (EMP) that provides affordable and quality essential medicines for the prevention and treatment of common diseases and health conditions [12]. Essential medicines are distributed through various channels, including public health facilities, private pharmacies, NGOs, and international donors. The government provides essential medicines free of charge to patients seeking treatment at public health facilities [12, 13], which are procured through a centralized system managed by the Department of Health Services. The distribution of essential medicines is facilitated by a network of warehouses and distribution centers throughout the country. However, despite all these initiatives, the country is still facing challenges to improve the quality of essential medicines.

The presence of substandard medicines in the pharmaceutical market of Nepal has been a concern for many years. Several studies and investigations have found evidence of substandard medicines being sold in Nepal [14, 15]. Studies have found substandard medicines in a range of therapeutic areas, including antibiotics, which can lead to treatment failure, drug resistance, and adverse effects. However, no studies have been found that assess the quality of essential medicines being supplied free of cost at the public health facilities of Nepal. Thus, this study aimed to assess the quality of selected essential medicines available in public health care facilities of Nepal.

## Methodology

A cross sectional descriptive design was used and the study was conducted in 21 districts of Nepal representing all seven provinces. Data and medicine sample collection was carried out from June to October, 2018 in 20 districts; while data collection in Mustang was possible only on April, 2019 due to unfavorable geographical conditions and transportation barriers. Altogether 63 health facilities were selected randomly comprising one Zonal / Regional/District Hospital, one PHCC and one HP from each selected district. However, one HP of Mustang district was dropped owing to unfavorable geographical condition during data collection. In total, 3 Zonal Hospitals, 1 Regional Hospital, 17 District Hospitals (DH), 21 PHCCs, 20 HPs and 5 Regional Medial Stores (RMSs) were included in the study. Districts were selected proportionately from all three ecological regions i.e. Terai, Hill and Mountain using lottery method. From each selected district, one Zonal / Regional/District Hospital, one Primary Health Care Center (PHCC) and one Health Post (HP) were identified using random number generator. Numbers of health facilities to include in the study was determined based on WHO guidelines on ‘how to investigate drug use in health facilities’ [16]. The WHO guidelines provide a step-by-step approach to investigating drug use in health facilities including designing study, collecting data, analyzing and reporting findings.

Face-to-face interview was taken with health facilities in-charge of selected health facilities regarding medicine procurement and storage. Structured questionnaire based on WHO guidelines- ‘how to investigate drug use in health facilities’ [16] was used to collect the information. To assess the storage condition of medicine store room i.e. protection from sunlight, humidity, heat, maintenance of cleanliness and ventilation, observation checklists were prepared based on Management Division guidelines [17]. The information was recorded based on observation. Digital device was used to record temperature and humidity of the storage room.

Altogether 20 molecules were selected from essential medicines list for this study. The selection criteria of medicines were made based on the therapeutic category and their used frequency. Medicines were selected such that the sample list includes all the major therapeutic category medicines which were considered to be commonly prescribed in various illnesses by the technical working team which includes members from regulatory authorities and different stakeholders. The list of selected molecules includes:

10 molecules from hospitals- Tinidazole 500 mg tablet, Ciprofloxacin 250 mg tablet, Paracetamol syrup 60 ml, Azithromycin 500 mg tablet, Iron supplement tablet, Povidone-iodine liquid 500 ml 5% w/v solution, Aluminium Hydroxide + magnesium hydroxide 250 mg tablet, Hyoscine butylbromide 10 mg tablet, Amlo225dipine 5 mg tablet, Metformin 500 mg tablet;
5 molecules from PHCCs- Cetrizine Hydrocloride 10 mg tablet, Metronidazole 400 mg tablet, Ciprofloxacin eye and ear drops, 5 ml 0.3%w/v, Sulfamethoxazole + trimethoprim tablet 960 mg DT, Fluconazole 150 mg tablet/capsule; 5 molecules from HPs- Amoxycillin capsule 500 mg, Ranitidine 150 mg tablet, Oral rehydration salt, Salbutamol 4 mg tablet, Paracetamol 500 mg tablet.

In addition to this, 6 molecules were collected from Regional Medical Stores (RMS) which included- Paracetamol 500 mg tablet, Tinidazole 500 mg tablet (130 tablets), Ciprofloxacin 250 mg tablet (130 tablets), Oral Rehydration Salts (25 sachets), Iron supplement (130 tablets) and Amlodipine 5 mg tablet (150 tablets). Medicine samples were collected on a zip lock bag with adequate label on it. In total, 244 batches of medicines were collected.

The drug samples collected from different health facilities were sent for testing to National Medicine Laboratory (NML)—the central laboratory that operates under the Ministry of Health and population of Nepal and is responsible for testing, analyzing, and verifying the quality, safety, and efficacy of various drugs, vaccines, and medical devices used in Nepal. The test results obtained from NML are deemed reliable and accurate as it regularly validates its analytical methods to meet international standards and guidelines.

The drug samples were tested for identification, weight variation, uniformity of content, dissolution, disintegration test, friability test, fill volume test, assay and PH based on the respective pharmacopeial guidelines that the drug sample followed. Drug samples were analyzed based on the pharmacopeial guidelines written on the label of the sample which included either United States Pharmacopeia(USP), Indian Pharmacopeia (IP) or British Pharmacopeia (BP).

The collected data were entered in epidata version 3.00 and Microsoft Excel 2007 before being analyzed with SPSS version 21.00. Ethical approval was obtained from Ethical Review Board of the Nepal Health Research Council (NHRC) prior to the onset of this study. Administrative approval was taken from Ministry of Health and Population (MoHP) to collect medicine samples from selected health facilities. An informed written consent was obtained before collection of data and medicine samples from health facilities in-charge of selected health facilities. A health facility in-charge is a health professional who is responsible for managing and overseeing the operation of the health facility including procurement of medicines, equipment, resource allocation, planning, coordination and reporting to the concerned authorities.

## Findings

Out of 244 batches of 20 generic medicines sent for in vitro analysis, 37 batches failed to comply the required pharmacopeal standard i.e. 15.2% of medicines were found substandard. Among the failed medicine samples, 23 (62.2%) batches of medicines were among the federal government supplied and 14 (37.4%) batches of medicine samples were procured by local health facilities. The list of substandard medicine samples included 4 batches of medicines (3 batch of ciprofloxacin eye/ear drop vial and 1 batch of ferrous sulphate with folic acid tablet) collected from RMS. The medicine samples were found substandard with respect to dissolution test, fill volume test, assay, friability test, uniformity of content and leakage test parameters. Table 1 demonstrates the list of substandard medicines along with their source of supply.

**Table 1:** List of identified substandard medicines.

Generic name	Collected from	Number(%) of failed batches	Remarks	Source of supply
Ciprofloxacin hydrochloride eye/ear drop	RMS	3(42.9)	Failed in Fill volume test	Federal Government
Iron supplements tablets	RMS and H	2(16.7)	One failed in uniformity of content and another one failed in both Content uniformity and assay	Federal Government
Metformin Hydrochloride tablets	H	5(29.4)	Failed in Dissolution	Local Government
Metronidazole Tablets	PHCC	12(60)	One failed in friability test three failed in dissolution	Local Government
			Eight failed in Dissolution	Federal Government
Paracetamol paediatric Oral suspension	HP	3(26.7)	Failed in Assay	Federal Government
Paracetamol tablets	H	3(13.6)	Failed in Dissolution	Local Government
Povidone-iodine solution	H	9(60)	Two failed in Leakage	Local Government
			Nine failed in Leakage	Federal Government
			One failed in Assay	Federal Government

The Table 2 depicts the storage condition of medicine store room of selected health facilities. The data shows that only 13% of health facilities, out of 62 selected health facilities, followed all the storage guideline of Management Division [17] for medicine storage i.e. protection from sunlight, humidity, heat and maintenance of good ventilation.

**Table 2:** Storage condition of medicine store room in selected Health facilities.

Variables	Hospitals(21)		PHCC(21)		HP(20)		Total(62)	
	N	%	N	%	N	%	N	%
Sunlight protection	9	42.9	5	23.8	10	50	24	38.7
Humidity protection	14	66.7	13	61.9	11	55	38	61.3
Heat protection	18	85.7	17	81	14	70	49	79.0
Good ventilation	17	81	18	85.7	15	75	50	80.6
All of the above	2	9.5	2	9.5	4	20	8	12.9

**Fig 1:**
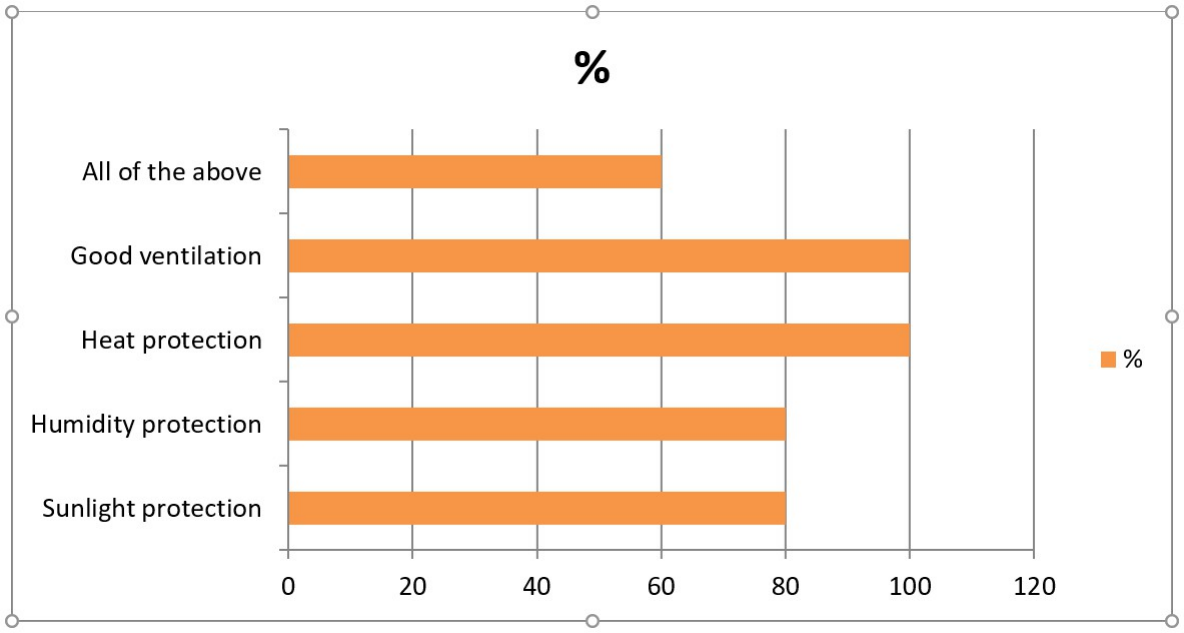
Storage condition of Regional Medical Stores.

Fig 1 illustrates the storage condition of medicine storeroom of five RMS. The data shows that all the recommended guidelines for medicine storage were found to be followed by 3 (60%) RMSs only.

The Table 3 demonstrates the range of temperature and humidity recorded at a point of time in the medicine store of health facilities. Out of total health facilities, maximum temperature record was found to be 37°C which is greater than the recommended temperature i.e 15°C to 25°C [18]. Likewise, among total health facilities, maximum humidity recorded was 86% which is also higher than the recommended humidity i.e. 60% or lower [18]. The data shows that temperature and humidity measure exceeded the recommended range for medicine storage in RMS also.

**Table 3:** Temperature and humidity records of medicine store room in Health Facilities and RMS.

	Hospitals (21)		PHCC (21)		HP (20)		RMS(5)	
	Min	Max	min	Max	Min	max	min	Max
Temperature	19°`C	37°C	20.9°C	34.6°C	23.7°C	37°C	30.5°C	37°C
Humidity	40%	77%	44%	86%	45%	80%	61%	77%

## Discussion

It has been found that about 15 percent of the 244 batches of essential medicines tested were identified as substandard. Only 13% of the 62 health facilities followed the drug storage guidelines. Moreover, the highest temperature and humidity record of the drug storage room among the selected health facilities were 37°C and 86 percent, respectively, which is greater than the recommended range. Despite the emphasis of Sustainable Development Goal (SDG) on “access to safe, effective, quality and affordable essential medicines and vaccines for all”, these findings are likely to put the SDG’s aim of Universal Health Coverage in jeopardy.

Section 12 of the Drug Act 1978 AD states that drugs meant for public consumption should be safe, efficacious and of quality standard complying the prescribed quality standard. The Act also prohibits on manufacture, sell, import, export or store of the drugs that do not adhere to the prescribed quality standard [19]. However, out of 244 batches of essential medicines tested for quality about 15% of medicines failed to meet the pharmacopeia requirement mentioned in their respective label hence found substandard. Substandard medicines are those medicines which fail to comply with the prescribed quality standard. Similar studies conducted in Nepal by Gyanwali P. et. al. [14] and Karki KB et al. [15] also identified 32.5% (out of 40 brands) and 4.21% (out of 214 samples) substandard drugs respectively in Nepal. Thus, the availability of substandard medicines in Nepal suggests for the improvement in the national regulatory system. This has prompted the need to review the prevailing law, regulation and policies and their implementation efficiency. Otherwise, the substandard quality of essential medicines even poses greater threat to public health and in achieving SDG goal including universal health coverage.

Evidences show that issues regarding substandard, spurious, falsely labeled, falsified and counterfeit (SSFFC) medicines have become pandemic in developing countries as well as in developed countries including India [3, 20–23]. The evidences reveal that India and China are the largest countries in manufacturing SSFC medicines [20, 24, 25]. As Nepal relies largely on neighboring countries India and China for import of medicine, the risk of SSFC medicines is inevitable in Nepal. A study carried out in Africa, Asia and South America identified both substandard and counterfeit medicines in Africa, Asia and South America with the highest number of counterfeit medicines in Asia [26]. The substandard medicines constituted 10.4% (out of 3,371 samples), 2.9% (out of 10,737 samples) and 11.5% (out of 955 samples) in Africa, Asia and South America respectively [26]. The therapeutic categories of those substandard medicines were: antimalarial, anti-tuberculosis, antibiotic, antiretroviral, anti-inflammatory and analgesics. A similar study carried out among seven countries of Africa and Asia, 21 substandard or falsified medicines out of 869 medicines sample were identified with anti-malarial drugs being greater in number [27]. Similarly, a systematic review on substandard and counterfeit medicines found that median prevalence of substandard or counterfeit medicines as 28.5% with greater prevalence of antimicrobials followed by anti-malarial and antibiotics [2].

The medicines, which were found substandard, were collected from public health care facilities of Nepal and they comprised medicines that are commonly used like: metronidazole, paracetamol, povidone iodine solution, ciprofloxacin drops, metformin hydrochloride and iron supplements. This scenario may not only decrease public trust towards health system, but could also cause financial loss and in some cases might exacerbate the disease condition.

Proper storage of medicines is vital to ensure the quality of the medicines. Protection from sunlight, humidity, excessive heat, temperature and maintenance of well ventilation are crucial to preserve the medicine quality. Poor storage condition might cause the degradation of the product. Despite policy statements and regulatory provision about storage [17], the storage of medicines seems to be largely ignored in Nepal. Only 13% (out of 62) of health facilities were found to protect medicines from sunlight, humidity and heat along with good ventilation based on medicine storage guidelines “Procurement handbook: storage and warehouse practice” of Management Division [17] in this study. Interestingly, all the medicine storage guidelines were found to be followed by Health posts in greater number compared to hospitals and Primary Health Care Centers. This might be because health posts in Nepal have fewer resources available to them compared to hospitals and primary health care centers which may make them more careful about how they store and manage medicines, as they cannot afford to waste resources on ineffective or expired medicines.

Health facilities of Nepal lacked infrastructures to maintain the constant recommended temperature i.e 15°C to 25°C and humidity (60% or lower) in the medicine storage room. However, other possible measures like protection from sunlight and direct exposure to floor and wall; which are prerequisites for medicine storage were found even not being followed in the health facilities including few RMS. The RMSs lacked in protecting medicines from sunlight and direct exposure to the floor. This highlights the greater negligence and weaknesses in pharmaceutical regulatory system and thus demands stringent rules and regulations in this sector. Likewise, recommended temperature and humidity for medicine storage room were found to have exceeded in health facilities and RMS due to lack of infrastructures. The situation can be worst in extreme weather condition i.e too hot and too cold leading to the degradation of the pharmaceutical product.

In addition to this, pharmaceutical companies may also affect the quality of medicines in a number of ways [28]. Firstly, if these companies do not adhere to good manufacturing practices (GMP), the quality of their products can be compromised. Poor quality raw materials or inadequate manufacturing processes can result in substandard medicines, which can be harmful to patients. Secondly, they may prioritize profits over quality, leading to the production and distribution of substandard or counterfeit medicines. Thirdly, they may lack the necessary resources or expertise to ensure that their products meet high quality standards. Finally, the supply chain for medicines can also impact the quality of medicines. If pharmaceutical companies do not adequately store or transport their products, the medicines can become degraded or contaminated, compromising their quality and effectiveness. Thus, it is important to monitor and regulate pharmaceutical companies to ensure the quality and safety of medicines as well.

In Nepal, there seems to be an increased attention towards the availability of essential medicines to the public from multiple sectors in past decades [29–31]. However, little effort is seen to ensure safety, efficacy and quality of the available essential medicines. The safety, efficacy and quality of essential medicines are of paramount important unlike the availability of essential medicines to the public because; poor quality of medicines can cause greater threat to the health and property of an individual.

The quality assurance systems for medicines in Nepal are still undergoing development and facing numerous challenges. The regulatory framework for medicines is not very strong, resulting in difficulty ensuring the safety, efficacy, and quality of medicines [28]. Additionally, the entities responsible for monitoring medicine quality may not have sufficient resources and expertise to perform their duties effectively. The supply chain for medicines in Nepal is complex and fragmented, leading to challenges in guaranteeing that essential medicines are being stored and transported correctly. This can cause medicines to deteriorate, thereby compromising their quality and effectiveness. Furthermore, testing capacities for medicines in Nepal are limited, and there may be insufficient trained personnel and equipment to evaluate medicine quality, which makes it difficult to detect substandard or counterfeit medicines that pose significant risks to patients [28]. Ultimately, these factors can negatively impact the quality of medicines being supplied in the country.

The study conducted was cross-sectional and could only provide a snapshot of the situation at a particular point in time. Furthermore, it was unable to establish a correlation between storage conditions and medicine quality. Thus, further studies are recommended to identify factors associated with substandard essential medicines in Nepal. In addition, addressing the existing challenges requires greater investment in the regulatory framework, with increased resources allocated to monitor and test essential medicines. There is also a need to strengthen the supply chain for medicines and raise public awareness about the significance of using high-quality medicines.

## Conclusion

A large number of essential medicines found in public health facilities are substandard, indicating a serious public health threat in Nepal. The storage of essential medicines in health facilities were found to be ignored in Nepal as all of the major procedures required for storing medicines in health facilities were found to be adopted by few health facilities only. Similarly, temperature and humidity records, both of which have a role in drug degradation, were found to be outside the acceptable range for medicine storage in various health facilities and RMS. Health facilities were found to be lacking in adequate infrastructure to sustain medicine storage conditions, which could be a contributing factor for substandard medicines in Nepal. Thus, all the infrastructures required for storage of medicines should be established and maintained in the health facilities of Nepal along with the provision to assess the quality of medicines supplied to them.

## Supporting information

S1 Data (XLSX)

S2 Data (XLSX)
